# Time to rethink colorectal cancer prevention strategies for lynch syndrome

**DOI:** 10.1186/s13053-024-00295-3

**Published:** 2025-02-07

**Authors:** Jan Lubinski, Rodney J. Scott

**Affiliations:** 1 Pomeranian Medical University, Szczecin, Poland; 2 University of Newcastle, Newcastle, NSW Australia

The adenoma carcinoma pathway described over 30 years ago [1] has aided significantly colonoscopic approaches to capturing colorectal adenomas before they transition into colorectal adenocarcinomas and become a much more serious health issue. The basis of screening is only beneficial if the sojourn time between screening is long enough to allow lesions to become apparent such that they that are captured before they become frank malignancies. This approach to colorectal cancer is predicated on the adenoma adenocarcinoma sequence first described by Fearon and Vogelstein (1990) and was based on the tumours not having a mutator phenotype (described now as microsatellite stable (MSS) disease) since the MSI phenotype was unknown at the time [1]. This model for MSS colorectal cancer has been shown to be beneficial for preventing the development of the majority of colorectal cancers in patients who are developing MSS tumours but like any model, it is only as good as the data used to derive it. Recent evidence casts some doubt on the magnitude of prevention offered by colonoscopy especially in relation to cancer related death [2].

With an increasing focus on disease development in Lynch Syndrome the adenoma/carcinoma model is a harder fit. In contrast to the majority of colorectal cancer patients, those that are associated with a deficiency in DNA mismatch repair (i.e. microsatellite instability (MSI)), tumour development appears to traverse a different route to malignant disease. In the report by Møller et al. (2024) using information from the Prospective Lynch Syndrome database (PLSD), ever shorter colonoscopic intervals do not change the incidence of colorectal cancer [3]. This is surprising but on reflection not unexpected. MSI predicts an accelerated rate of tumour development (i.e. the mutator phenotype) [4] and, as a consequence, the sojourn time between disease initiation and the appearance of malignancy is much shorter than that observed in MSS tumours.

In the intervening years since the genetic basis of LS was described it has become apparent that LS associated tumours are much more immunogenic and as such, more readily succumb to the effects of immune surveillance [5]. Since LS tumours generate frameshift peptides that are recognised and cleared presumably during the process of immune surveillance. Since this is not a perfect system, it is not surprising that tumours occasionally rapidly develop and acquire a variety of mutations in genes that promote colorectal tumorigenesis [6, 7]. This information strongly suggests a different paradigm needs to be established to reconcile the absence of effect of increasing colonic surveillance but not decreasing the frequency of colorectal cancer. In the report by Møller et al. (2024) the difference between MSI associated colorectal tumours and the more common MSS tumours is made primarily by the absence of change in cancer incidence even when the colonoscopic screening interval is reduced from once every 3 years to annually [3].

There remains much to learn about tumour development in LS and its relationship with environmental factors that either promote or hinder disease development. Overall, we have perhaps been falling into the trap of genetic determinism when thinking about mutations in genes that predispose to malignancy and have assumed that there is only one road that leads to Rome.
